# Effects of 12-week supervised combined aerobic and resistance training on body composition and physical function in overweight or obese adults with multiple cardiometabolic risk factors: a randomized trial

**DOI:** 10.3389/fpubh.2026.1896850

**Published:** 2026-08-19

**Authors:** Jineng Chen, Junlin Chen, Kailin Mao, Zhejun Chen, Zepeng Lin

**Affiliations:** 1 School of Physical Education, Sanming University, Sanming, Fujian, China; 2 Quanzhou Huaguang Vocational College, Quanzhou, Fujian, China; 3 School of Physical Education, Huaqiao University, Quanzhou, Fujian, China; 4 School of Information Engineering, Sanming University, Sanming, Fujian, China; 5 School of Physical Education, Putian University, Putian, Fujian, China

**Keywords:** aerobic exercise, body composition, cardiometabolic risk, hospital-based exercise, physical function, randomized trial, resistance training

## Abstract

**Background:**

Combined aerobic and resistance training is recommended for adults with cardiometabolic risk factors, but evidence from hospital-based integrated sports and health service settings remains limited.

**Objective:**

This 12-week randomized trial evaluated the effects of supervised combined aerobic and resistance training on body composition and physical function in overweight or obese adults with multiple cardiometabolic risk factors.

**Methods:**

Adults with overweight or obesity and at least one additional clinically diagnosed cardiometabolic risk factor were randomized in a planned 2:1 ratio to intervention or control using a computer-generated stratified permuted-block sequence with allocation concealment. The intervention group completed supervised exercise three times weekly for 12 weeks. Each 60 min session included warm-up, aerobic exercise, resistance exercise, and flexibility/cool-down, with intensity monitored using heart rate, heart-rate reserve, perceived exertion, symptoms, and tolerance. Controls maintained usual lifestyle and dietary habits and received weekly telephone follow-up. Co-primary outcomes were whole-body fat mass and handgrip strength. Baseline-adjusted linear mixed-effects models estimated week 12 between-group differences.

**Results:**

Of 60 individuals screened, 52 were randomized, and all completed week 12 assessment. At week 12, the intervention group had greater reductions in whole-body fat mass than controls (adjusted difference, −1.48 kg; 95% CI, −1.85 to −1.11; Holm-adjusted *P* < 0.001) and greater improvement in handgrip strength (1.16 kg; 95% CI, 0.56 to 1.77; Holm-adjusted *P* < 0.001). Exploratory secondary analyses with unadjusted *P* values suggested improvements in body weight, body mass index, body fat percentage, trunk fat mass, lower-limb explosive power, 4-m gait speed, one-leg standing balance, and sit-and-reach flexibility. Trunk muscle mass and whole-body response time were not statistically significant. Mean attendance was 86.7%, with no serious adverse events.

**Conclusion:**

In this single-center randomized trial, 12-week supervised combined aerobic and resistance training was associated with modest improvements in fat-related body composition and selected physical-function outcomes in overweight or obese adults with multiple cardiometabolic risk factors. Cardiometabolic biomarkers were not reassessed at follow-up, and findings should be interpreted as preliminary.

## Introduction

Transparent reporting of randomized trials and adequate description of interventions are essential for interpreting and replicating exercise intervention studies (1, 2). Multiple cardiometabolic risk factors, including hypertension, hyperglycemia, dyslipidemia, and overweight or obesity, commonly cluster in middle-aged and older adults and are associated with elevated risk of cardiovascular disease, diabetes, functional decline, and loss of independence (3–8). Lifestyle intervention is a central component of risk management because it can target adiposity, metabolic regulation, vascular function, and physical capacity simultaneously (9–13). Exercise is particularly relevant in this population because low cardiorespiratory fitness and impaired muscle function may reinforce cardiometabolic vulnerability and limit daily activity (14, 15).

Aerobic exercise and resistance training have complementary physiological effects. Aerobic exercise can increase energy expenditure and support reductions in fat mass, whereas resistance training can improve strength, neuromuscular function, and mobility-related performance (16–21). Contemporary exercise guidance therefore often emphasizes combined aerobic and muscle-strengthening activity for adults and for people with cardiometabolic risk (9–13). However, many community and clinical exercise programs are insufficiently described for replication, and there remains a need for pragmatic randomized evidence from integrated sports and health service settings in which exercise professionals and medical staff jointly supervise participants.

This randomized trial examined the effects of a 12-week supervised combined aerobic and resistance training program on body composition and physical function in overweight or obese adults with multiple cardiometabolic risk factors at Sanming Shaxian District General Hospital, Fujian Province, China. The co-primary outcomes were whole-body fat mass and handgrip strength, representing fat-related body composition and muscle function. We hypothesized that the intervention would reduce whole-body fat mass and improve handgrip strength compared with usual lifestyle control. Because cardiometabolic biomarkers were not reassessed at follow-up, the trial was not designed to determine changes in blood pressure, glycemic control, or lipid profiles.

## Methods

### Study design and setting

This was a 12-week randomized trial conducted in a hospital-based integrated sports and health service setting at Sanming Shaxian District General Hospital, Fujian Province, China. Reporting was guided by the principles of CONSORT 2025 for randomized trials. Intervention details are reported according to the Template for Intervention Description and Replication (TIDieR) framework in Supplementary Table S1 (1, 2).

### Participants

Participants were overweight or obese adults with at least one additional cardiometabolic risk factor. Eligibility was assessed before randomization. Written informed consent was obtained from all participants before enrollment. The study was approved by the Ethics Committee of Sanming Shaxian District General Hospital (approval No. Lunshen [2025] No. 002) and was conducted in accordance with the Declaration of Helsinki.

### Eligibility criteria

Eligible participants were adults receiving health management services at Sanming Shaxian District General Hospital who had overweight or obesity plus at least one additional cardiometabolic risk factor, including hypertension, hyperglycemia, or dyslipidemia. Overweight was defined as a BMI of 24.0 to < 28.0 kg/m^2^, and obesity as a BMI ≥28.0 kg/m^2^ according to Chinese adult criteria. Abdominal obesity was defined as waist circumference ≥90 cm in men or ≥85 cm in women. Hypertension was defined as systolic blood pressure ≥140 mmHg, diastolic blood pressure ≥90 mmHg, a previous physician diagnosis, or current use of antihypertensive medication. Hyperglycemia was defined as fasting plasma glucose ≥6.1 mmol/L, HbA1c ≥5.7%, a previous physician diagnosis of diabetes or impaired glucose regulation, or current use of glucose-lowering medication or insulin. Dyslipidemia was defined as triglycerides ≥1.7 mmol/L, total cholesterol ≥5.2 mmol/L, LDL-C ≥3.4 mmol/L, low HDL-C (< 1.0 mmol/L in men or < 1.3 mmol/L in women), a previous physician diagnosis, or current use of lipid-lowering medication. Participants also had to provide written informed consent and be willing to complete baseline, week 8, and week 12 assessments.

Participants were excluded if medical staff judged that they had contraindications to exercise, unstable cardiovascular disease, uncontrolled hypertension, severe musculoskeletal or neurological conditions limiting exercise, severe hepatic or renal disease, acute infection or inflammatory disease, current structured exercise training, inability to complete follow-up, or other conditions considered unsafe for participation in supervised exercise.

### Randomization and blinding

A computer-generated random allocation sequence was prepared in R by an independent statistician who was not involved in participant recruitment, intervention delivery, outcome assessment, or data analysis. A stratified permuted-block randomization method was used with a planned 2:1 allocation ratio favoring the intervention group. Randomization was stratified by sex and cardiometabolic risk-factor burden, with randomly varying block sizes of 3 and 6. Cardiometabolic risk-factor burden was categorized as one vs. two or more additional metabolic risk factors beyond overweight/obesity. Allocation assignments were placed in sequentially numbered, opaque, sealed envelopes. After written informed consent, eligibility confirmation, and baseline assessment were completed, the next envelope in sequence was opened by a research coordinator who was not involved in outcome assessment. The final allocation resulted in 34 participants in the intervention group and 18 in the control group. Due to the behavioral nature of the exercise intervention, participants and exercise instructors could not be blinded to group allocation. Outcome assessors were blinded to group allocation. The primary statistical analyses were conducted by the study analyst using a dataset in which group allocation was coded as Group A and Group B. The group labels were decoded only after the primary analyses of the co-primary outcomes had been completed.

### Intervention

Participants in the intervention group attended supervised combined aerobic and resistance training three times per week for 12 weeks, with each session lasting 60 min. Each session consisted of a 5 min warm-up, 30 min of aerobic exercise, 20 min of resistance exercise, and 5 min of flexibility and cool-down activities. The intervention was delivered at Sanming Shaxian District General Hospital and jointly supervised by exercise professionals and medical staff. Detailed session-level exercise prescription is provided in Supplementary Table S1.

Exercise risk assessment and intensity adjustment were guided by the chronic disease management system, heart-rate monitoring, heart-rate reserve (HRR), rating of perceived exertion (RPE), symptoms, movement quality, and participant tolerance. Heart rate, perceived exertion, and symptoms were monitored during sessions, and exercise intensity was dynamically adjusted by exercise professionals and medical staff. Heart-rate reserve was calculated as estimated maximal heart rate minus resting heart rate, and target heart rate was calculated as resting heart rate plus the prescribed percentage of heart-rate reserve. Estimated maximal heart rate was calculated as 220 minus age.

Formal one-repetition maximum (1RM) testing was not performed for safety and feasibility reasons. Resistance intensity was prescribed using RPE, movement quality, and participant tolerance. Exercise professionals instructed exercise technique, adjusted exercise intensity, and recorded attendance. Medical staff monitored symptoms and exercise safety. Participants could stop exercise immediately if they experienced discomfort. Carbohydrate snacks or glucose water were available for participants at risk of hypoglycemia. Exercise adherence was calculated as the percentage of supervised sessions attended out of the 36 planned sessions. Participants who completed at least 80% of the planned sessions, corresponding to at least 29 sessions, were considered adherent.

### Control group

Participants in the control group maintained their usual lifestyle and dietary habits and did not receive structured exercise training or an individualized exercise prescription. Weekly telephone follow-up lasted approximately 5–10 min and was used to monitor physical condition, physical activity, dietary habits, daily activity levels, and adverse events. Telephone follow-up did not provide individualized exercise coaching. Participants were asked not to initiate new regular moderate-to-vigorous exercise programs during the trial.

### Outcome measures

The co-primary outcomes were whole-body fat mass (kg) and handgrip strength (kg). Secondary body composition outcomes were body weight (kg), body mass index (BMI; kg/m^2^), body fat percentage (%), whole-body muscle mass (kg), trunk fat mass (kg), and trunk muscle mass (kg). Secondary physical function outcomes were whole-body response time (s), lower-limb explosive power score (device-specific units), 4-m gait speed (m/s), one-leg standing balance on the left and right sides (s), and sit-and-reach flexibility (cm).

Body composition was assessed using an InBody 770 bioelectrical impedance analyzer (InBody Co., Ltd., Seoul, Republic of Korea). Measurements were performed in the morning, and participants were instructed to fast overnight for at least 8 h before testing. Participants were instructed to avoid vigorous exercise, alcohol, and excessive caffeine intake for 24 h before testing, to avoid large fluid intake immediately before testing, to empty the bladder before measurement, and to remove shoes, socks, and metal objects. Participants wore light clothing, and whenever possible each participant was assessed at the same time of day by the same trained assessor using the same device. Whole-body fat mass, body fat percentage, whole-body muscle mass, trunk fat mass, and trunk muscle mass were recorded for analysis. Bioelectrical impedance analysis is widely used in clinical and field settings but is sensitive to measurement conditions and should be interpreted accordingly (22–24).

Handgrip strength was measured using a handgrip dynamometer. Both hands were tested twice, with a 1 min rest interval, and the average of the best left- and right-hand values was used for analysis. The analysis variable was expressed in kg. Handgrip strength is a practical indicator of muscle strength and physical function in clinical and epidemiological research (25, 26).

Whole-body response time was measured using a response-time device. Participants stood on a sensing mat and jumped vertically as quickly as possible after seeing a red visual signal. Five practice trials were performed, followed by five recorded responses; the mean recorded response time was used for analysis. This measure reflects a combined reaction and movement response rather than pure reaction time.

Lower-limb explosive power was assessed using a Leg Power device. Participants sat on the testing chair with the back supported, safety belt fastened, hands holding the handles, and feet on the footplate. Participants pushed the footplate as forcefully as possible by extending the knees. Four trials were performed with 30 s rest intervals, and the best value was used for analysis. The device output was recorded as a device-specific score; therefore, lower-limb explosive power is reported in device-specific units and should be interpreted as within-device change rather than as a directly comparable physiological power value.

Gait speed was assessed on a 6-m walkway marked at 0, 1, 5, and 6 m. The time between the 1-m and 5-m marks was recorded, and gait speed was calculated as 4 m divided by walking time. Gait speed is a well-established indicator of mobility and physical function (27, 28). Eyes-closed one-leg standing balance was assessed separately for the left and right sides. Each side was tested twice, and the best value was used for analysis. Sit-and-reach flexibility was assessed using an automatic sit-and-reach device. Two trials were performed, and the best value was used for analysis. Best valid attempts were used for these field-based physical fitness tests after standardized instructions and practice.

### Sample size

No formal *a priori* sample-size calculation was performed for this trial.

The sample size was determined by the number of eligible participants recruited from the hospital-based health management service during the predefined study period. Therefore, the study may have had limited statistical power, particularly for secondary and non-significant outcomes.

### Statistical analysis

Statistical analyses were performed using R version 4.6.0 with lme4, lmerTest, and emmeans. Continuous variables were summarized as mean ± standard deviation, and categorical variables as counts and percentages. The co-primary outcomes were whole-body fat mass and handgrip strength. For each outcome, baseline-adjusted linear mixed-effects models were fitted using follow-up values at week 8 and week 12 as dependent variables. Fixed effects included group, time, group-by-time interaction, the baseline value of the corresponding outcome, age, and sex, with participant ID included as a random intercept. A participant-level random intercept was used to account for within-participant correlation across follow-up assessments; no random slopes or additional residual covariance structure were specified. The primary estimand was the adjusted between-group difference at week 12. Estimated marginal means and model-based between-group contrasts were obtained using the emmeans package. Holm correction was applied only to the two co-primary outcomes. No composite ‘both-or-either' success rule was used; the two co-primary outcomes were interpreted as separate co-primary endpoints after Holm correction. *P* values for secondary outcomes were not adjusted for multiplicity and were interpreted as exploratory. Analysis of covariance (ANCOVA) models using week 12 outcomes adjusted for baseline values, age, and sex were conducted as sensitivity analyses. Two-sided *P* values < 0.05 were considered statistically significant.

## Results

### Participant flow and baseline characteristics

A total of 60 individuals were screened for eligibility. Eight individuals were excluded, and 52 participants were randomized, with 34 allocated to the intervention group and 18 allocated to the control group. All randomized participants completed the week 12 assessment and were included in the final analysis. Complete follow-up was supported by flexible assessment scheduling and telephone reminders before follow-up visits. The analysis dataset contained 156 observations from 52 participants, and all participants had measurements at three time points.

Baseline characteristics are shown in Table 1. The overall mean age was 59.15 ± 6.22 years. The control group included 7 men and 11 women, and the intervention group included 14 men and 20 women. Baseline BMI was 27.87 ± 2.52 kg/m^2^ in the control group and 27.75 ± 1.78 kg/m^2^ in the intervention group.

**Table 1:** Baseline characteristics.

Variable	Overall (*n* = 52)	Control (*n* = 18)	Intervention (*n* = 34)
Age, years	59.15 ± 6.22	59.28 ± 7.71	59.09 ± 5.40
Sex: Male	21 (40.4%)	7 (38.9%)	14 (41.2%)
Sex: Female	31 (59.6%)	11 (61.1%)	20 (58.8%)
Height, cm	163.27 ± 7.30	160.77 ± 7.38	164.60 ± 7.00
Body weight, kg	74.23 ± 8.48	72.15 ± 8.61	75.33 ± 8.32
BMI, kg/m^2^	27.79 ± 2.04	27.87 ± 2.52	27.75 ± 1.78
Systolic blood pressure, mmHg	137.94 ± 12.00	137.17 ± 12.97	138.35 ± 11.63
Diastolic blood pressure, mmHg	89.10 ± 7.79	88.83 ± 7.20	89.24 ± 8.19
Baseline cardiometabolic biomarkers			
Fasting plasma glucose (FPG), mmol/L	6.42 ± 1.18	6.39 ± 1.21	6.44 ± 1.17
Glycated hemoglobin (HbA1c), %	6.13 ± 0.72	6.10 ± 0.76	6.15 ± 0.70
Triglycerides (TG), mmol/L	2.06 ± 0.78	2.10 ± 0.81	2.04 ± 0.77
Total cholesterol (TC), mmol/L	5.28 ± 0.86	5.31 ± 0.89	5.26 ± 0.85
LDL-C, mmol/L	3.24 ± 0.69	3.27 ± 0.72	3.22 ± 0.68
HDL-C, mmol/L	1.14 ± 0.24	1.13 ± 0.25	1.15 ± 0.24
Whole-body fat mass, kg	25.71 ± 4.59	25.59 ± 6.07	25.78 ± 3.69
Body fat percentage, %	34.67 ± 5.30	35.19 ± 6.47	34.39 ± 4.66
Whole-body muscle mass, kg	31.12 ± 4.40	29.51 ± 2.72	31.98 ± 4.89
Handgrip strength, kg	26.05 ± 6.48	24.96 ± 7.40	26.63 ± 5.98
Lower-limb explosive power score, device-specific units	87.15 ± 17.80	79.72 ± 17.42	91.09 ± 16.95
4-m gait speed, m/s	1.07 ± 0.11	1.06 ± 0.12	1.07 ± 0.10
Sit-and-reach flexibility, cm	9.09 ± 5.03	10.63 ± 5.00	8.28 ± 4.92
Cardiometabolic risk factors			
Overweight, *n* (%)	33 (63.5%)	12 (66.7%)	21 (61.8%)
Obesity, *n* (%)	19 (36.5%)	6 (33.3%)	13 (38.2%)
Abdominal obesity, *n* (%)	41 (78.8%)	14 (77.8%)	27 (79.4%)
Hypertension, *n* (%)	35 (67.3%)	12 (66.7%)	23 (67.6%)
Hyperglycemia, *n* (%)	28 (53.8%)	10 (55.6%)	18 (52.9%)
Dyslipidemia, *n* (%)	31 (59.6%)	11 (61.1%)	20 (58.8%)
One additional metabolic risk factor, *n* (%)	13 (25.0%)	5 (27.8%)	8 (23.5%)
Two additional metabolic risk factors, *n* (%)	22 (42.3%)	8 (44.4%)	14 (41.2%)
Three additional metabolic risk factors, *n* (%)	17 (32.7%)	5 (27.8%)	12 (35.3%)
≥2 additional metabolic risk factors, *n* (%)	39 (75.0%)	13 (72.2%)	26 (76.5%)
Medication use			
Antihypertensive medication, *n* (%)	24 (46.2%)	8 (44.4%)	16 (47.1%)
Glucose-lowering medication, *n* (%)	15 (28.8%)	5 (27.8%)	10 (29.4%)
Insulin, *n* (%)	3 (5.8%)	1 (5.6%)	2 (5.9%)
Lipid-lowering medication, *n* (%)	18 (34.6%)	6 (33.3%)	12 (35.3%)
Statins, *n* (%)	15 (28.8%)	5 (27.8%)	10 (29.4%)
Diuretics, *n* (%)	5 (9.6%)	2 (11.1%)	3 (8.8%)
Weight-loss medication, *n* (%)	0 (0.0%)	0 (0.0%)	0 (0.0%)
Systemic corticosteroids, *n* (%)	0 (0.0%)	0 (0.0%)	0 (0.0%)
Any cardiometabolic medication, *n* (%)	32 (61.5%)	11 (61.1%)	21 (61.8%)

Values are mean ± standard deviation or *n* (%). Additional metabolic risk factors refer to hypertension, hyperglycemia, and dyslipidemia beyond overweight or obesity. Statins were reported as a subgroup of lipid-lowering medications. Insulin use was reported separately and was included within glucose-lowering medication use.

Baseline cardiometabolic biomarker values were broadly comparable between groups. In the control and intervention groups, fasting plasma glucose was 6.39 ± 1.21 and 6.44 ± 1.17 mmol/L, HbA1c was 6.10 ± 0.76% and 6.15 ± 0.70%, triglycerides were 2.10 ± 0.81 and 2.04 ± 0.77 mmol/L, total cholesterol was 5.31 ± 0.89 and 5.26 ± 0.85 mmol/L, LDL-C was 3.27 ± 0.72 and 3.22 ± 0.68 mmol/L, and HDL-C was 1.13 ± 0.25 and 1.15 ± 0.24 mmol/L, respectively.

Baseline cardiometabolic biomarker values, risk-factor distribution, and medication characteristics are also shown in Table 1. All participants had overweight or obesity. Hypertension, hyperglycemia, and dyslipidemia were common at baseline, and most participants had two or more additional metabolic risk factors beyond overweight or obesity. Baseline use of antihypertensive, glucose-lowering, and lipid-lowering medications was broadly comparable between groups.

### Adherence and safety

In the intervention group, participants completed a mean of 31.2 ± 3.1 of the 36 planned supervised sessions, corresponding to an average attendance rate of 86.7%. Overall, 29 of 34 participants (85.3%) completed at least 80% of the planned sessions. During the intervention period, two participants reported mild transient muscle soreness and one participant experienced dizziness during training, which resolved after rest. No severe hypoglycemic episodes, cardiovascular events, exercise-related injuries requiring medical treatment, or withdrawals due to adverse events were reported.

### Effects on co-primary outcomes

At week 12, the intervention group showed a greater reduction in whole-body fat mass than the control group, with an adjusted between-group difference of −1.48 kg (95% CI, −1.85 to −1.11; *P* < 0.001; Holm-adjusted *P* < 0.001). Handgrip strength also improved more in the intervention group than in the control group, with an adjusted between-group difference of 1.16 kg (95% CI, 0.56 to 1.77; *P* < 0.001; Holm-adjusted *P* < 0.001). Directionally similar effects were present at week 8 for both co-primary outcomes. Detailed adjusted estimates for body composition and physical function outcomes are presented in Tables 2, 3.

**Table 2:** Effects on body composition outcomes.

Outcome	Week 8 adjusted difference (95% CI)	*P* value	Week 12 adjusted difference (95% CI)	*P* value	Holm-adjusted *P*
Whole-body fat mass, kg	−0.56 (−0.93 to −0.19)	0.004	−1.48 (−1.85 to −1.11)	< 0.001	< 0.001
Body weight, kg	−0.94 (−1.30 to −0.58)	< 0.001	−1.60 (−1.95 to −1.24)	< 0.001	—
BMI, kg/m^2^	−0.37 (−0.51 to −0.23)	< 0.001	−0.61 (−0.75 to −0.47)	< 0.001	—
Body fat percentage, %	−0.74 (−1.14 to −0.34)	< 0.001	−1.11 (−1.51 to −0.71)	< 0.001	—
Whole-body muscle mass, kg	0.19 (−0.09 to 0.47)	0.189	0.31 (0.03 to 0.59)	0.029	—
Trunk fat mass, kg	−0.34 (−0.63 to −0.06)	0.018	−0.60 (−0.89 to −0.32)	< 0.001	—
Trunk muscle mass, kg	0.13 (−0.10 to 0.35)	0.274	0.12 (−0.10 to 0.35)	0.278	—

Adjusted differences are intervention minus control. Models were adjusted for baseline outcome value, age, and sex, with participant ID included as a random intercept. Holm correction was applied only to the two co-primary outcomes. *P* values for secondary outcomes were not adjusted for multiplicity and should be interpreted as exploratory.

**Table 3:** Effects on physical function outcomes.

Outcome	Week 8 adjusted difference (95% CI)	*P* value	Week 12 adjusted difference (95% CI)	*P* value	Holm-adjusted *P*
Handgrip strength, kg	0.92 (0.31 to 1.53)	0.004	1.16 (0.56 to 1.77)	< 0.001	< 0.001
Whole-body response time, s	−0.00 (−0.02 to 0.02)	0.759	−0.02 (−0.04 to 0.00)	0.052	—
Lower-limb explosive power score, device-specific units	4.45 (2.19 to 6.71)	< 0.001	7.04 (4.78 to 9.30)	< 0.001	—
4-m gait speed, m/s	0.01 (−0.01 to 0.03)	0.165	0.04 (0.02 to 0.06)	< 0.001	—
One-leg standing balance, left, s	0.16 (−0.85 to 1.17)	0.756	2.12 (1.11 to 3.14)	< 0.001	—
One-leg standing balance, right, s	1.81 (0.90 to 2.71)	< 0.001	1.62 (0.72 to 2.53)	< 0.001	—
Sit-and-reach flexibility, cm	0.80 (−0.01 to 1.61)	0.052	1.77 (0.97 to 2.58)	< 0.001	—

Adjusted differences are intervention minus control. Models were adjusted for baseline outcome value, age, and sex, with participant ID included as a random intercept. Holm correction was applied only to the two co-primary outcomes. *P* values for secondary outcomes were not adjusted for multiplicity and should be interpreted as exploratory.

### Effects on body composition outcomes

For body composition outcomes, adjusted between-group differences favored the intervention group primarily for fat-related measures. At week 12, adjusted between-group differences favored the intervention group for body weight (−1.60 kg; 95% CI, −1.95 to −1.24; *P* < 0.001), BMI (−0.61 kg/m^2^; 95% CI, −0.75 to −0.47; *P* < 0.001), body fat percentage (−1.11 percentage points; 95% CI, −1.51 to −0.71; *P* < 0.001), and trunk fat mass (-0.60 kg; 95% CI, −0.89 to −0.32; *P* < 0.001). Whole-body muscle mass showed a modest adjusted difference at week 12 (0.31 kg; 95% CI, 0.03 to 0.59; *P* = 0.029), whereas trunk muscle mass did not differ significantly between groups (0.12 kg; 95% CI, −0.10 to 0.35; *P* = 0.278).

### Effects on physical function outcomes

Physical function outcomes generally favored the intervention group. At week 12, adjusted between-group differences were 7.04 device-specific units for lower-limb explosive power score (95% CI, 4.78 to 9.30; *P* < 0.001), 0.041 m/s for 4-m gait speed (95% CI, 0.023 to 0.059; *P* < 0.001), 2.12 s for left one-leg standing balance (95% CI, 1.11 to 3.14; *P* < 0.001), 1.62 s for right one-leg standing balance (95% CI, 0.72 to 2.53; *P* < 0.001), and 1.77 cm for sit-and-reach flexibility (95% CI, 0.97 to 2.58; *P* < 0.001). Whole-body response time did not differ statistically significantly between groups at week 12 (−0.021 s; 95% CI, −0.043 to 0.0002; *P* = 0.052).

### Sensitivity analyses and figure summary

**Figure 1:**
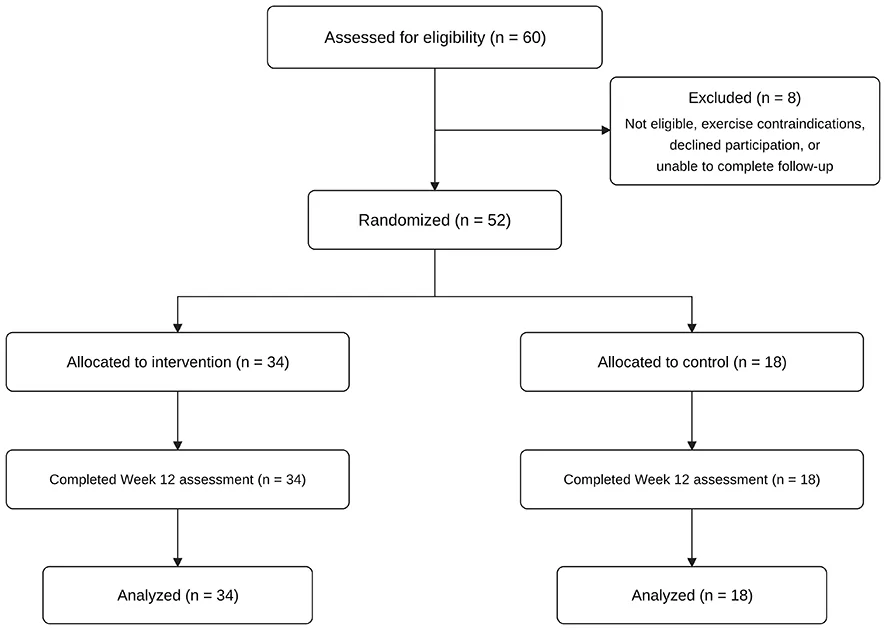
CONSORT flow diagram. The diagram shows screening, randomization, allocation, week 12 completion, and analysis populations.

**Figure 2:**
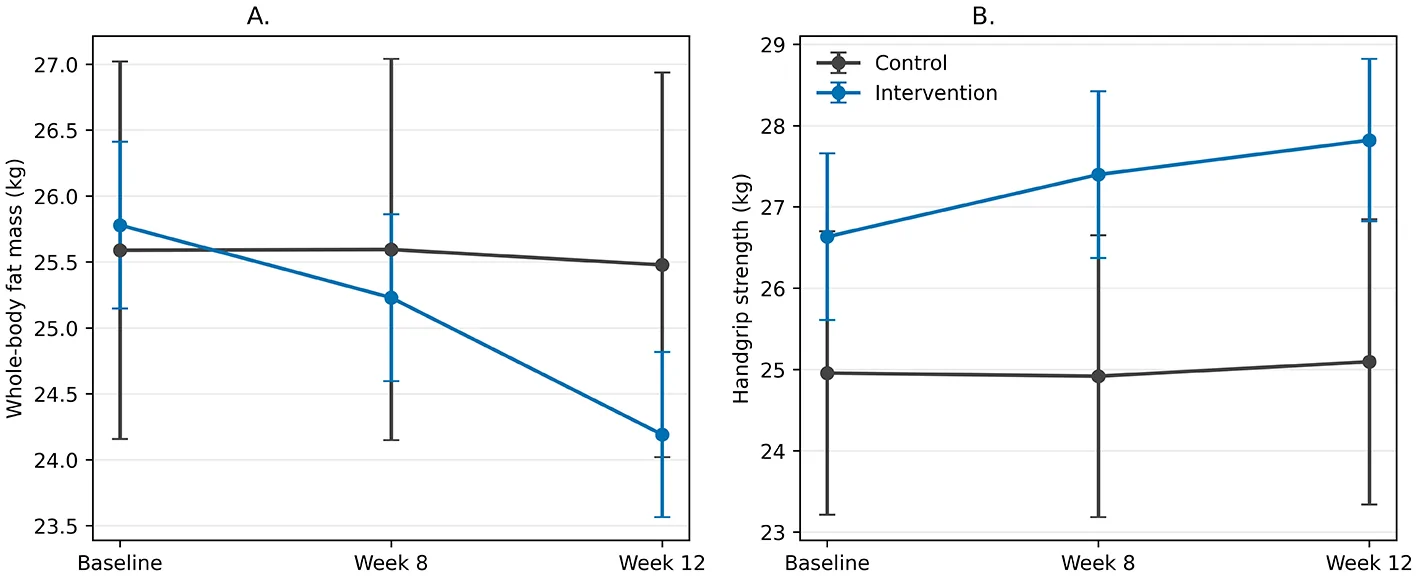
Time trends of co-primary outcomes. Values represent mean ± standard error at baseline, week 8, and week 12 in the intervention and control groups. **(A)** Whole-body fat mass. **(B)** Handgrip strength.

**Figure 3:**
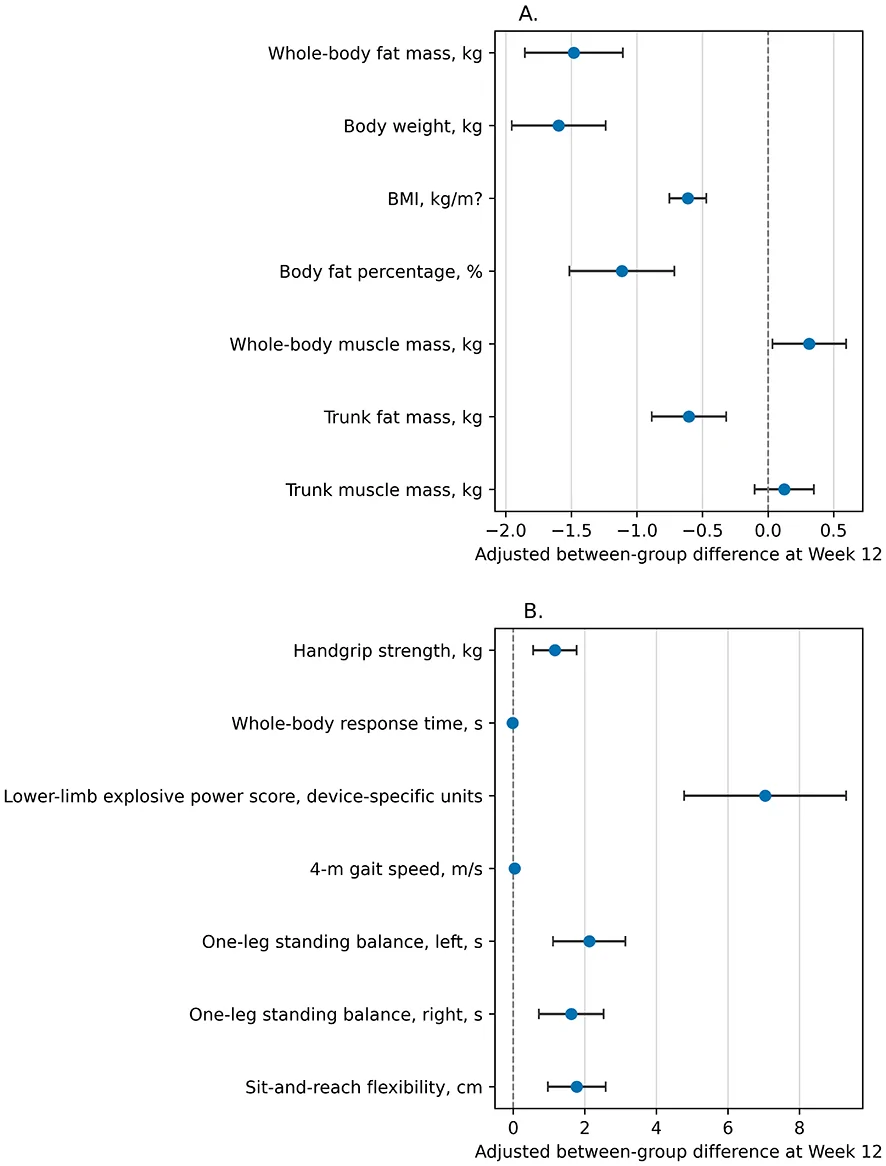
Adjusted between-group differences in body composition and physical function outcomes at week 12. Points represent baseline-adjusted between-group differences between the intervention and control groups at week 12, and horizontal bars represent 95% confidence intervals. Models were adjusted for baseline outcome value, age, and sex, with participant ID included as a random intercept. Negative values indicate greater reductions in the intervention group, whereas positive values indicate greater increases in the intervention group. **(A)** Body composition outcomes. **(B)** Physical function outcomes.

ANCOVA sensitivity analyses using week 12 outcomes adjusted for baseline values, age, and sex were directionally consistent with the primary mixed-effects analyses for the co-primary outcomes and most exploratory secondary outcomes (Supplementary Table S2). Whole-body muscle mass was less stable in the sensitivity analysis, supporting cautious interpretation of muscle mass findings. The CONSORT flow diagram summarizes screening, allocation, follow-up, and analysis (Figure 1). Time trends for the co-primary outcomes are shown in Figure 2, and adjusted week 12 between-group differences are summarized in Figure 3.

## Discussion

### Principal findings

In this 12-week randomized trial conducted in a hospital-based integrated sports and health service setting, supervised combined aerobic and resistance training was associated with modest reductions in whole-body fat mass and small improvements in handgrip strength in overweight or obese adults with multiple cardiometabolic risk factors. Exploratory secondary outcomes suggested additional improvements in body weight, BMI, body fat percentage, trunk fat mass, lower-limb explosive power, gait speed, balance, and flexibility. In contrast, effects on trunk muscle mass and whole-body response time were not statistically significant, and the evidence for increased muscle mass was modest and less consistent. Because cardiometabolic biomarkers were not reassessed at follow-up, these findings should not be interpreted as evidence of improved cardiometabolic risk status.

### Comparison with previous research

The pattern of findings is consistent with previous guidance and evidence indicating that combined aerobic and resistance exercise can improve adiposity-related and functional outcomes, while effects on cardiometabolic biomarkers require direct assessment (16–21, 29, 30). The observed fat-related changes align with the expected effects of repeated aerobic exercise combined with supervised training volume, while improvements in handgrip strength and lower-limb performance are compatible with the neuromuscular benefits of resistance training. Handgrip strength is widely used as a practical marker of muscle strength and health-related function, supporting its selection as a co-primary functional outcome in this trial (25, 26). The present study adds pragmatic evidence from a hospital-based integrated service model, where exercise professionals and medical staff jointly supervised participants.

### Possible mechanisms

Several mechanisms may explain the observed benefits. Aerobic exercise may increase energy expenditure and fat oxidation, contributing to reductions in whole-body and trunk fat mass. Resistance training may improve neuromuscular recruitment, motor coordination, and force production, which can translate into better handgrip strength, explosive power, gait speed, balance, and flexibility-related performance even when changes in measured muscle mass are modest. The combined format may therefore be particularly suitable for adults with multiple cardiometabolic risk factors because it addresses both adiposity and physical function. However, the present trial did not reassess blood pressure, glucose, HbA1c, or lipid outcomes at follow-up, so mechanistic interpretations related to cardiometabolic risk reduction remain speculative.

### Clinical and public health implications

The high attendance rate and absence of serious adverse events support the short-term feasibility and safety of supervised combined training in this setting. A hospital-based integrated sports and health service model may provide a practical pathway for delivering structured exercise to overweight or obese adults with cardiometabolic risk factors, especially when medical staff can help monitor symptoms and exercise professionals can individualize training intensity. However, the absolute changes observed in whole-body fat mass, handgrip strength, and gait speed were modest. Therefore, the findings support emphasizing short-term fat-related body composition improvement and selected functional gains as realistic goals, while avoiding claims of clinically meaningful functional enhancement or cardiometabolic risk reduction.

### Strengths and limitations

Strengths of this study include its randomized design, complete week 12 follow-up, supervised intervention delivery, clearly defined co-primary outcomes, baseline-adjusted mixed-effects modeling, and ANCOVA sensitivity analyses. The intervention prescription is reported using the TIDieR framework and includes session structure, aerobic and resistance modes, intensity targets, progression rules, supervision, adherence criteria, and safety procedures.

This study has several limitations. First, the trial was not prospectively registered, which limits independent verification of outcome prespecification and the analysis plan. Therefore, the findings should be interpreted as preliminary rather than confirmatory. Second, no formal *a priori* sample-size calculation was performed for this trial, and the modest sample size may have limited statistical power, particularly for exploratory secondary and non-significant outcomes. Third, although a planned 2:1 randomization scheme and allocation concealment were implemented, the single-center hospital-based design and unequal group sizes may limit precision and generalizability. Fourth, the control group was not attention-matched to the supervised exercise intervention; therefore, between-group differences may partly reflect differential contact time, supervision, expectancy, or Hawthorne effects rather than the exercise stimulus alone. Fifth, participants and exercise instructors could not be blinded, although outcome assessors were blinded to group allocation and the study analyst used masked group labels until the primary analyses of the co-primary outcomes had been completed. Sixth, although participants had multiple cardiometabolic risk factors at baseline, blood pressure, glucose, HbA1c, and lipid outcomes were not reassessed at follow-up, so the trial cannot determine whether the intervention improved cardiometabolic risk status. Seventh, body composition was assessed using bioelectrical impedance analysis rather than a reference imaging method and may be influenced by hydration and testing conditions despite standardized procedures. Finally, secondary outcomes were exploratory and not adjusted for multiplicity, and the use of best-attempt scores for some field-based physical fitness tests may have increased measurement variability compared with averaged trials. In addition, physical activity and dietary intake outside the supervised sessions were not objectively measured or fully controlled.

## Conclusion

In this single-center randomized trial, a 12-week supervised combined aerobic and resistance training program delivered in a hospital-based integrated sports and health service setting was associated with modest reductions in fat-related body composition and small improvements in selected physical-function outcomes among overweight or obese adults with multiple cardiometabolic risk factors. The intervention showed acceptable short-term adherence and safety. However, because the trial was not prospectively registered, used a modest sample, lacked an attention-matched control group, and did not reassess cardiometabolic biomarkers at follow-up, the findings should be interpreted as preliminary.

## Data Availability

The datasets presented in this article are not readily available because they contain de-identified health-management and medication-related information subject to institutional and privacy requirements. Requests to access the datasets should be directed to Zepeng Lin, zepenglin47@gmail.com. The randomization log, allocation-concealment records, and de-identified baseline clinical and medication data are retained by the study team and can be made available to the editor upon reasonable request, subject to institutional and privacy requirements.
