# Supplementary material for: Effects of 12-week supervised combined aerobic and resistance training on body composition and physical function in overweight or obese adults with multiple cardiometabolic risk factors: a randomized trial
Source: Front Public Health. 2026 Aug 19;14:1896850. doi: 10.3389/fpubh.2026.1896850 (PMC13534076; doi:10.3389/fpubh.2026.1896850)
Supplement: Supplementary file 1 [file Table_1.docx]

**Supplementary Table 1. Session-level exercise prescription according to the TIDieR framework.**

| Item | Description |
| --- | --- |
| Intervention period | 12 weeks. |
| Frequency | Three sessions per week. |
| Total sessions | 36 sessions. |
| Session duration | 60 minutes per session. |
| Session structure | 5-minute warm-up + 30 minutes of aerobic exercise + 20 minutes of resistance exercise + 5 minutes of flexibility/cool-down activities. |
| Setting | Hospital-based integrated sports and health service exercise-intervention site at Sanming Shaxian District General Hospital. |
| Supervisors | Exercise professionals provided training instruction; medical staff provided safety monitoring. |
| Warm-up | Low-intensity walking, shoulder and neck mobility, hip/knee/ankle dynamic mobility, and light dynamic stretching. |
| Aerobic modes | Brisk walking, cycle ergometer, low-impact aerobic exercise, or step training, selected according to individual ability. |
| Aerobic intensity | Weeks 1-4: 50%-60% HRR or RPE 11-12; Weeks 5-8: 60%-70% HRR or RPE 12-13; Weeks 9-12: 65%-75% HRR or RPE 13-14. |
| HRR calculation and target heart-rate formula | Heart-rate reserve was calculated as estimated maximal heart rate minus resting heart rate. Target heart rate was calculated as resting heart rate plus the prescribed percentage of heart-rate reserve. Estimated maximal heart rate was calculated as 220 minus age. |
| Resistance modes | Elastic bands, freehand/body-weight exercises, and light equipment. |
| Resistance exercises | Sit-to-stand training or squats, calf raises, elastic-band rowing, chest press, shoulder press, biceps curls, triceps extensions, glute bridge, or hip abduction. |
| Sets and repetitions | Weeks 1-4: 1-2 sets of 12-15 repetitions; Weeks 5-8: 2 sets of 10-15 repetitions; Weeks 9-12: 2-3 sets of 8-12 repetitions. |
| Rest intervals | 60-90 seconds between sets. |
| Resistance intensity | RPE 12-15, provided that movement quality was maintained and no obvious discomfort occurred. |
| Progression rules | If participants could easily complete the target repetitions for two consecutive sessions with RPE <12, elastic-band resistance, light-equipment load, or one additional set was added. |
| Flexibility/cool-down | Static stretching of the hamstrings, quadriceps, calves, chest/shoulders, and lower back, 15-30 seconds per movement. |
| Heart-rate monitoring | Heart-rate devices were worn throughout each session; maximum and average heart rate during training were recorded. |
| RPE monitoring | RPE was recorded once after the aerobic component and once after the resistance component. |
| Blood pressure monitoring | Blood pressure was measured before training and rechecked after training when necessary. |
| Stopping criteria | Chest pain, marked dyspnea, dizziness, palpitations, abnormal blood pressure, hypoglycemic symptoms, or worsening joint pain. |
| Hypoglycemia plan | Biscuits, sugar water, or glucose water were available on site. |
| Adherence definition | Completion of at least 80% of sessions, corresponding to at least 29 of 36 sessions. |
| Actual adherence | Mean 31.2 ± 3.1 sessions; attendance rate 86.7%; 29 of 34 participants achieved at least 80% attendance. |
| Safety/adverse events | Two cases of mild transient muscle soreness and one case of dizziness during training that resolved after rest; no serious adverse events. |

**Supplementary Table 2. Analysis of covariance (ANCOVA) sensitivity analyses.**

| Outcome | **Week 12 adjusted difference (95% CI)** | P value |
| --- | --- | --- |
| Whole-body fat mass, kg | -1.48 (-1.88 to -1.07) | <0.001 |
| Handgrip strength, kg | 1.12 (0.43 to 1.80) | 0.002 |
| Body weight, kg | -1.59 (-1.98 to -1.20) | <0.001 |
| BMI, kg/m² | -0.61 (-0.76 to -0.46) | <0.001 |
| Body fat percentage, % | -1.12 (-1.57 to -0.66) | <0.001 |
| Whole-body muscle mass, kg | 0.30 (-0.04 to 0.63) | 0.084 |
| Trunk fat mass, kg | -0.61 (-0.93 to -0.28) | <0.001 |
| Trunk muscle mass, kg | 0.07 (-0.21 to 0.36) | 0.600 |
| Lower-limb explosive power score, device-specific units | 7.30 (4.91 to 9.69) | <0.001 |
| 4-m gait speed, m/s | 0.04 (0.02 to 0.06) | <0.001 |
| One-leg standing balance, left, s | 2.10 (0.91 to 3.30) | <0.001 |
| One-leg standing balance, right, s | 1.61 (0.64 to 2.59) | 0.002 |
| Sit-and-reach flexibility, cm | 1.74 (0.82 to 2.66) | <0.001 |
| Whole-body response time, s | -0.02 (-0.05 to 0.00) | 0.095 |

Note. Values are adjusted between-group differences (intervention minus control) with 95% confidence intervals. ANCOVA models were adjusted for baseline outcome value, age, and sex. P values for secondary outcomes were not adjusted for multiplicity and should be interpreted as exploratory. ANCOVA, analysis of covariance; CI, confidence interval.
